# Supplementary material for: DisguisOR: holistic face anonymization for the operating room
Source: Int J Comput Assist Radiol Surg. 2023 May 23;18(7):1209–15. doi: 10.1007/s11548-023-02939-6 (PMC10329611; doi:10.1007/s11548-023-02939-6)
Supplement: Supplementary file 1 — (pdf 4693 KB) [file 11548_2023_2939_MOESM1_ESM.pdf]

DisguisOR – Supplementary Materials

Lennart Bastian*, Tony Danjun Wang*, Tobias Czempiel, Benjamin Busam and Nassir Navab

Computer Aided Medical Procedures, Technical University Munich, Munich, Germany.

* Equal Contribution. Contact: {first}.{last}@tum.de.

1 Methods

1.1 Obstruction Detection

In Figure 1, we detail how we handle obstructions that occur in the back-projection of the 3D face mesh. If an object is positioned between the face and a camera then the calculated distance between the camera and face in 3D will be larger than the distance inferred from the depth. Specifically, we obtain the 2D coordinates of 20 fixed vertices of the 3D face mesh for each camera view. Using the depth map, we calculate the corresponding 3D coordinates using the depth values of each 2D coordinate. If the distance between a 3D coordinate inferred from the depth map and the corresponding actual 3D coordinate of a vertex is similar, we conclude the face is visible for the camera in question.

1.2 Dataset Curation

In Figure 2, we provide a set of examples of the ground truth annotations in our dataset. It denotes the annotation bounding boxes of obstructed faces and cases where no face information is visible.

1.3 3D Key-Point Smoothing

To generate less noisy and more robust keypoints we perform a 3D keypoint smoothing and tracking over an entire sequence before fitting the SMPL mesh onto the 3D keypoints. The smoothing process helps to diminish outliers caused by incorrect triangulations during the 3D human pose estimation stage and to interpolate missed human poses. This allows for a more reliable human

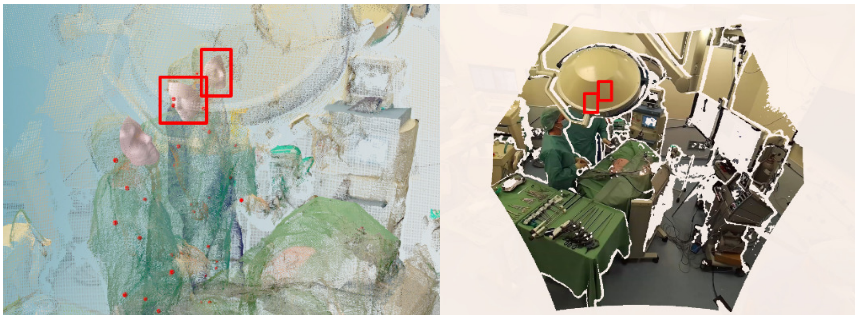

Fig. 1 Obstructed faces in the point cloud and color image. The left image shows three face meshes in 3D, the right image shows the depth map of surgical camera 2. The two red rectangles reveal the positions of two face meshes in both images. Our method checks for obstructions by calculating the distance between the meshes' 3D locations and the inferred 3D locations from the depth map.

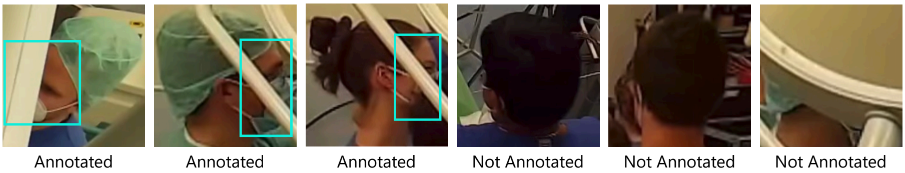

Fig. 2 Examples of faces from our dataset with their respective annotation

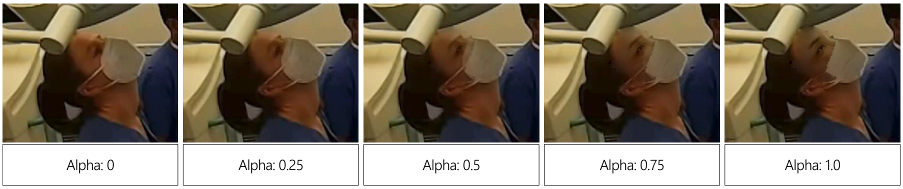

Fig. 3 Examples of tuning the parameter for image blending with poisson image editing. With an alpha value of 1.0, no gradient of the background image is used, while a value of 0 uses only the gradients of the background image. We chose 0.725 as parameter to balance image quality and sufficient anonymization.

mesh regression onto the 3D human poses. Furthermore, the tracking enables anonymizing each person with the same face texture in every frame.

1.4 Rendering: Poisson Image Editing

We further highlight the effect of different blending parameters of the poisson image editing image harmonization (Figure 3). As an additional robustness towards privacy preservation, one may also opt to blur in the source image prior to blending (Figure 4) or avoid blending altogether (Figure 3 Alpha: 1.0).

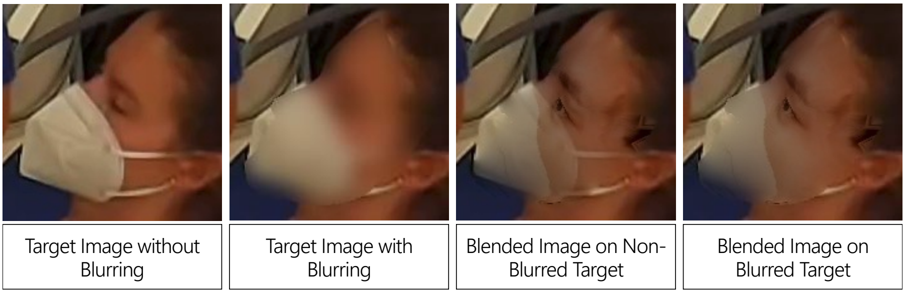

Fig. 4 Illustration of combining poisson image editing with blurring. Blurring the target image allows to further hide prominent background information in the result. The medical mask's contour of the original target blends seamlessly with the final output~~.~~

2 Results and Discussion

2.1 Face Localization

In Tables 1, 2 and 3, we present the precision (P), recall (R), F1-score (F1), and number of annotated faces of each individual camera and scenario. The precision of DisguisOR is lower for some scenarios than that of DSFD, notably in the surgical cameras (SC1 and SC2). This is mainly due to the hardware constraints of the Kinect camera system, which does not generate a corresponding depth value for each pixel. Cropping to the depth field of view and downsizing the color image to the resolution of the depth camera would mitigate these issues and likely favor DisguisOR – however, we perform inference for all methods at native resolution of 2048x1536. While the self-supervised domain adapation (SSDA) [1] with DSFD increased the recall significantly, especially in the surgical cameras, it also decreased the precision, resulting in a lower F1-score.

Table 1 Comparison of precision (P), recall (R) and F1-score (F1) at IOU@0.4 of DSFD [2], the Self-Supervised Domain Adaption (SSDA) method of [1], and DisguisOR on the **Easy Scenario** across all cameras. The last column depicts the number of ground truth faces in each camera view~~.~~

	DSFD [2]			SSDA [1]			DisguisOR			No. Faces
	P	R	F1	P	R	F1	P	R	F1	
SC1	90.5	91.9	91.2	44.8	98.4	61.6	83.5	91.1	87.1	384
SC2	87.2	87.8	87.5	71.6	91.1	80.2	75.7	86.5	80.7	327
WFC1	69.3	86.8	77.1	51.0	91.1	65.4	61.8	84.2	71.3	190
WFC2	97.8	98.3	98.1	62.2	97.6	76.0	87.3	92.2	89.7	409
Avg.	86.2	91.2	88.5	57.4	94.6	70.8	77.1	88.5	82.2	327

2.1.1 Different Confidence Thresholds

~~In~~ Table 4 depicts the precision, recall and F1-scores of DSFD [2] using different confidence thresholds. The default confidence threshold is 0.5. We average each metric across all four cameras, and present the results for each scenario.

Table 2 Comparison of precision (P), recall (R) and F1-score (F1) at IOU@0.4 of DSFD [2], the Self-Supervised Domain Adaption (SSDA) method of [1], and DisguisOR on the **Medium Scenario** across all cameras. The last column depicts the number of ground truth faces in each camera view.

	DSFD [2]			SSDA [1]			DisguisOR			No. Faces
	P	R	F1	P	R	F1	P	R	F1	
SC1	97.5	84.2	90.4	71.9	93.4	81.3	87.4	97.3	92.1	558
SC2	63.4	37.9	47.4	29.6	53.5	38.1	35.1	78.5	48.5	256
WFC1	87.2	96.2	91.5	67.1	88.3	76.3	71.7	79.8	75.5	426
WFC2	94.0	96.0	95.0	94.0	95.4	94.7	93.3	92.3	92.8	1077
Avg.	85.5	78.6	81.1	65.7	82.7	72.6	71.9	87.0	77.2	579

Table 3 Comparison of precision (P), recall (R) and F1-score (F1) at IOU@0.4 of DSFD [2], the Self-Supervised Domain Adaption (SSDA) method of [1], and DisguisOR on the **Hard Scenario** across all cameras. The last column depicts the number of ground truth faces in each camera view.

	DSFD [2]			SSDA [1]			DisguisOR			No. Faces
	P	R	F1	P	R	F1	P	R	F1	
SC1	88.2	16.9	28.4	10.3	52.8	17.2	19.6	97.8	32.7	89
SC2	88.3	55.2	67.9	89.6	74.6	81.4	62.3	74.1	67.7	232
WFC1	76.2	87.1	81.3	73.5	56.4	63.8	51.1	79.7	62.3	202
WFC2	99.2	95.3	97.2	73.2	88.5	80.1	93.8	90.8	92.3	763
Avg.	88.0	63.6	68.7	61.7	68.1	60.7	56.7	85.6	63.7	321

Lowering the confidence scores increases the recall, but at a significant cost of precision. Even at lower confidence thresholds, DSFD does not attain the recall of DisguisOR. Furthermore, errant detections cover large swaths of the images (Figure 5). Subsequent anonymization steps could impact the image integrity and affect downstream tasks.

Table 4 Comparison of precision (P), recall (R) and F1-score (F1) at IOU@0.4 of DSFD [2] with different confidence thresholds and DisguisOR on all scenarios. P, R and F1 are averaged across all cameras.

	DSFD [2] @ 0.1			DSFD [2] @ 0.025			DisguisOR		
	P	R	F1	P	R	F1	P	R	F1
Easy	73.3	94.8	81.7	12.5	96.5	20.6	77.1	88.5	82.2
Medium	71.2	84.0	76.4	26.9	88.4	38.3	71.9	87.0	77.2
Hard	64.1	69.6	64.8	23.6	73.6	28.9	56.7	85.6	63.7

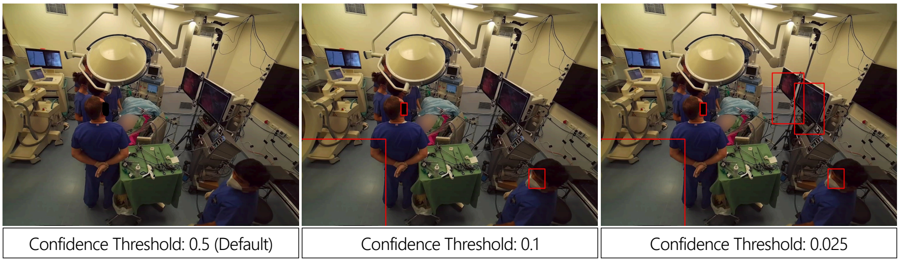

Fig. 5 Detections of DSFD [2] with different confidence thresholds. While lowering the threshold of DSFD leads to additional correct predictions, this typically comes at the expense of errant predictions which cover large portions of the image.

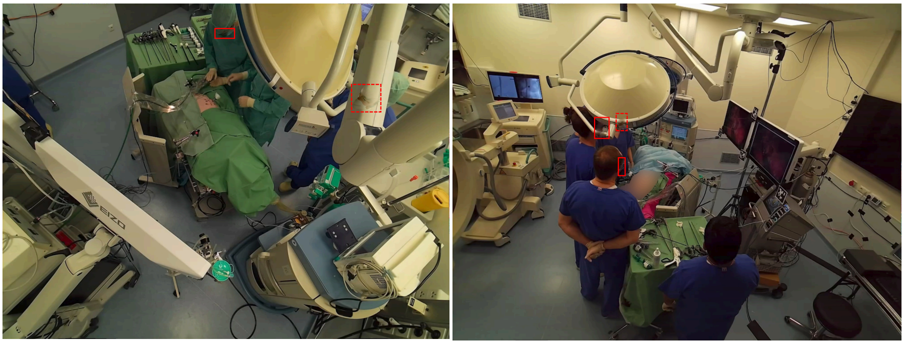

Fig. 6 Illustration of two different frames, each with a false positive detection due to a failed occlusion check (best viewed digitally). Dotted red face bounding boxes denote false positive detections, while solid bounding boxes depict true positive detections. Many DisguisOR false positive predictions are due to failed occlusion checks. However, these artifacts represent a small area of the image.

2.2 Experiments on Downstream Tasks

Face Detection. To evaluate the preservation of image integrity, we compare our anonymization method with the three conventional anonymization methods (blurring, pixelization and blackening) and DeepPrivacy [3] on downstream face detection. This experiment is designed to measure the degree to which an anonymization method creates unnatural alterations, which would lead to errant predictions from an existing method. We compare the performance of the pre-trained DSFD [2] model on images from all three scenarios anonymized through several methods. For a fair comparison between the two methods, we only consider face detections made by both DeepPrivacy and DisguisOR. We report the percentage of average precision (AP) at an intersection over union of greater than 0.4 (IOU@0.4) retained with respect to the original unaltered images.

Human Pose Estimation. In order to further demonstrate the image quality preservation of our method, we evaluate the widely used AlphaPose [4] human pose estimator, pre-trained on COCO [5], to understand the effect of unnatural image artifacts on human pose estimation. We generate pseudo-ground truth

by projecting the 3D joint positions of each generated SMPL mesh into each camera view. In accordance with previously established works [1], we use the percentage of correct keypoints (PCK) metric to measure how many keypoints were accurately predicted within a threshold. DeepPrivacy is omitted from this experiment to avoid an unfair bias in favor of DisguisOR due to how pseudo-ground truth is generated.

3 Results.

Face Detection. Table 5 depicts the downstream face detection performance of the face detector DSFD [2] on images anonymized through various obfuscation methods. We report the percentage AP retained compared to the AP achieved on the original unaltered images. The results show that blackening the face area removes a considerable amount of information, making it prohibitively difficult for a method to localize a face. Blurring or pixelization is less detrimental to the detection methods' performance. Nevertheless, the detection results of both methods are severely impacted by the blurring of face regions. This becomes even more evident in difficult scenarios. For all three conventional anonymization approaches, the performance drop is severe, and the AP for the face detection task is too low for most use cases. In contrast, our proposed method generates faces that the detection methods can identify with high accuracy.

In the easy scenario, we observe a decrease of merely 2% in DSFD's face detection AP, highlighting the preservation qualities of our method. DeepPrivacy is able to retain more AP in the medium and hard scenarios, most likely since it generates synthetic faces with distinct facial features. Using a maskless face texture further increases the retained AP by enabling face detectors to rely on more facial elements, such as the nose and mouth. However, this comes at a cost of image quality, as apparent by the experiments in the main paper. ~~These results indicate that a given template and source image harmonization may be more suitable for a certain application.~~ These results indicate that the realistic faces generated by DisguisOR could mitigate costly annotation and retraining due to an inferior anonymization method. Furthermore, they also indicate that a given template and source image harmonization may be more suitable for a certain application.

Human Pose Estimation. In Table 6 we report the PCK of the human pose estimator AlphaPose [4] on differently anonymized frames. Human pose estimators are less susceptible to limited modifications of the face area. Therefore, the deviation of the PCKs is less severe. Blurring, pixelization, and blackening of the faces can confuse the methods resulting in a decreased performance. It is interesting to see that the anonymization limited to the region of the head still has a measurable negative impact on the joint detection of the hip. This again highlights the importance of retaining the information and the need to anonymize without severe information loss. The PCK on images anonymized by our method is the highest, demonstrating the realism of our method.

Table 5 Percentage of face detection AP of DSFD [2] at IOU@0.4 for differently anonymized images with the AP on original images as baseline. Masked Texture denotes that blended templates contain medical masks (see Figure 3), whereas Maskless Texture denotes that blended templates do not contain medical masks.

Anonymization Method	Easy Scenario	Medium Scenario	Hard Scenario
Blackening	12.2	10.9	9.1
Gaussian Blur	18.4	19.3	7.8
Pixelization	72.4	60.6	63.1
DeepPrivacy [3]	99.1	**98.9**	**94.2**
DisguisOR (Masked Texture)	98.2	85.3	74.7
DisguisOR (Maskless Texture)	**100.0**	92.8	82.8

Table 6 PCKh@0.5 results for AlphaPose [4] on differently anonymized images. Images are taken from all scenarios across all cameras.

Anonymization Method	Head	Shoulder	Elbow	Wrist	Hip	Knee	Ankle	Avg.
Blackening	58.8	76.1	71.6	63.7	66.7	31.4	25.7	56.4
Pixelization	71.1	79.0	74.5	65.9	72.2	32.5	26.1	61.4
Gaussian Blur	72.6	81.5	76.8	67.3	74.2	**34.1**	26.3	63.0
DisguisOR	**78.2**	**82.0**	**77.4**	**67.4**	**76.2**	33.9	**27.0**	**65.0**

4 Appendix

For reproducibility, we provide the GitHub repository link and python version for each method that we have used in this paper. All computations were performed on a computer with 64GB of RAM and an NVIDIA GeForce RTX 2080 Ti. DeepPrivacy needed approximately 2.44s for anonymizing each frame (i.e., 4 images),while DisguisOR needed approximately 6.47s for ~~anonymizing~~ each frame ~~(i.e., 4 images)~~ (see Table 7). The majority of this time is spent on point cloud alignment and rendering, which could be made more efficient. More specifically, it needed 0.52s for 2D human pose estimation, 0.23 for 3D human pose estimation, 0.62s for human mesh estimation and 3.74s for registration and 1.36s rendering. The memory footprint of DeepPrivacy reached around 4.6GB, while DisguisOR used approximately 6.5GB.

Table 7 The runtime for each frame (i.e., 4 images) of DisguisOR split into respective stages.

Stage	Runtime (seconds)
2D Human Pose Estimation	0.52
3D Human Pose Esimtation	0.23
Human Mesh Estimation	0.62
Point Cloud Registration	3.74
Rendering	1.36
Total	6.47

- DisguisOR https://github.com/wngTn/disguisor (Python 3.7.2)
  - DEKR [6] https://github.com/HRNet/DEKR (Python 3.7.2)
  - VoxelPose [7] https://github.com/microsoft/voxelpose-pytorch (Python 3.10.2)
  - EasyMocap [8] https://github.com/zju3dv/EasyMocap (Python 3.6.2)
  - SMPL [9] https://smpl.is.tue.mpg.de
- Evaluation code versions
  - DSFD [2] https://github.com/hukkelas/DSFD-Pytorch-Inference (Python 3.8.13)
  - DeepPrivacy [3] https://github.com/hukkelas/DeepPrivacy (Python 3.8.2)
  - AlphaPose [4] https://github.com/MVIG-SJTU/AlphaPose (Python 3.7.2)

References

[1] Issenhuth, T., Srivastav, V., Gangi, A., Padoy, N.: Face detection in the operating room: Comparison of state-of-the-art methods and a self-supervised approach. CoRR **abs/1811.12296** (2018)

[2] Li, J., Wang, Y., Wang, C., Tai, Y., Qian, J., Yang, J., Wang, C., Li, J., Huang, F.: DSFD: dual shot face detector. CoRR **abs/1810.10220** (2018)

[3] Hukkelås, H., Mester, R., Lindseth, F.: Deepprivacy: A generative adversarial network for face anonymization. CoRR **abs/1909.04538** (2019)

[4] Fang, H., Xie, S., Lu, C.: RMPE: regional multi-person pose estimation. CoRR **abs/1612.00137** (2016)

[5] Lin, T., Maire, M., Belongie, S.J., Bourdev, L.D., Girshick, R.B., Hays, J., Perona, P., Ramanan, D., Dollár, P., Zitnick, C.L.: Microsoft COCO: common objects in context. CoRR **abs/1405.0312** (2014)

[6] Geng, Z., Sun, K., Xiao, B., Zhang, Z., Wang, J.: Bottom-up human pose estimation via disentangled keypoint regression. CoRR **abs/2104.02300** (2021)

[7] Tu, H., Wang, C., Zeng, W.: End-to-end estimation of multi-person 3d poses from multiple cameras. CoRR **abs/2004.06239** (2020)

[8] EasyMoCap - Make human motion capture easier. Github (2021). https://github.com/zju3dv/EasyMocap

[9] Loper, M., Mahmood, N., Romero, J., Pons-Moll, G., Black, M.J.: SMPL:

A skinned multi-person linear model. ACM Trans. Graphics (Proc. SIGGRAPH Asia) **34**(6), 248–124816 (2015)
